# Human Observers and Automated Assessment of Dynamic Emotional Facial Expressions: KDEF-dyn Database Validation

**DOI:** 10.3389/fpsyg.2018.02052

**Published:** 2018-10-26

**Authors:** Manuel G. Calvo, Andrés Fernández-Martín, Guillermo Recio, Daniel Lundqvist

**Affiliations:** ^1^Department of Cognitive Psychology, Universidad de La Laguna, San Cristóbal de La Laguna, Spain; ^2^Instituto Universitario de Neurociencia (IUNE), Universidad de La Laguna, Santa Cruz de Tenerife, Spain; ^3^Department of Health Sciences, Universidad Internacional de la Rioja, Logroño, Spain; ^4^Institute of Psychology, Universität Hamburg, Hamburg, Germany; ^5^Department of Clinical Neuroscience, Karolinska Institutet, Stockholm, Sweden

**Keywords:** facial expression, dynamic, action units, KDEF, FACET

## Abstract

Most experimental studies of facial expression processing have used static stimuli (photographs), yet facial expressions in daily life are generally dynamic. In its original photographic format, the Karolinska Directed Emotional Faces (KDEF) has been frequently utilized. In the current study, we validate a dynamic version of this database, the KDEF-dyn. To this end, we applied animation between neutral and emotional expressions (happy, sad, angry, fearful, disgusted, and surprised; 1,033-ms unfolding) to 40 KDEF models, with morphing software. Ninety-six human observers categorized the expressions of the resulting 240 video-clip stimuli, and *automated face analysis* assessed the evidence for 6 expressions and 20 facial action units (AUs) at 31 intensities. Low-level image properties (luminance, signal-to-noise ratio, etc.) and other purely perceptual factors (e.g., size, unfolding speed) were controlled. Human recognition performance (accuracy, efficiency, and confusions) patterns were consistent with prior research using static and other dynamic expressions. Automated assessment of expressions and AUs was sensitive to intensity manipulations. Significant correlations emerged between human observers’ categorization and automated classification. The KDEF-dyn database aims to provide a balance between experimental control and ecological validity for research on emotional facial expression processing. The stimuli and the validation data are available to the scientific community.

## Introduction

Research on facial expression processing (see reviews in Nelson and Russell, 2013; Calvo and Nummenmaa, 2016) has generally utilized static faces as stimuli, obtained from standardized databases such as the Pictures of Facial Affect (PoFA; Ekman and Friesen, 1976), the Karolinska Directed Emotional Faces (KDEF; Lundqvist et al., 1998), the NimStim Stimulus Set (Tottenham et al., 2002), the Radboud Faces Database (RaFD; Langner et al., 2010), FACES (Ebner et al., 2010) and others (for a review and evaluation, see Cowie et al., 2005; Anitha et al., 2010; Sandbach et al., 2012). Yet, in social encounters and face-to-face communication, facial expressions are generally dynamic. Further, research has shown that motion benefits affect recognition (see Krumhuber et al., 2013; Calvo et al., 2016; Wingenbach et al., 2016). Accordingly, it is important to use dynamic stimuli for investigating recognition of facial expressions.

A number of *dynamic* expression databases have been developed, generally involving on-line video recordings of facial activity, which represent a valuable advance (e.g., van der Schalk et al., 2011; Banziger et al., 2012; Kaulard et al., 2012; Zhang et al., 2014; O’Reilly et al., 2016; Wingenbach et al., 2016). Krumhuber et al. (2017) have reviewed and discussed the major issues of 22 dynamic expression databases. In the current study, the proposal of a new stimulus set (KDEF-dyn) aims to make a contribution by taking two issues into account. First, the control of possible *perceptual confounds* with non-expressive factors that may affect expression recognition. They involve low-level image properties of the stimuli, such as illumination and light source, size of the face relative to the background, head-face orientation, or changes in facial appearance like hair, make up, eyeglasses, jewelry, etc. They may be difficult to control for in video-recordings of spontaneous expressions. Yet, to unequivocally attribute emotion recognition to facial expression *per se*, all the facial stimuli across types of expressions must be comparable on these non-expressive factors. Further, the control of such factors may be critical for paradigms using neurophysiological (such as event-related potentials, ERPs; see Naples et al., 2015) or eyetracking (e.g., probability of first fixation in a particular face region, or pupillometry; e.g., Calvo and Nummenmaa, 2011) measures, which are particularly sensitive to physical image properties. To this end, all the face stimuli in our KDEF-dyn set are standardized in size, resolution, location, and frontal view, in addition to multiple low-level image properties (luminance, contrast, etc.).

A second issue is concerned with the objective validation of expressions and component facial actions across *multiple intensities*. According to Valstar et al. (2015, 2017), many existing benchmark databases show expressions at fixed intensities (generally, the apex or maximum intensity) or do not support the evaluation of intensity effects. Computational algorithms have been developed to automatically detect Facial Action Coding System (FACS) action units (AUs; Ekman et al., 2002), which are anatomical changes in the facial morphology that can be associated to specific emotions (e.g., AU12 or lip corner puller, to happiness; or AU4, brow lowerer, to anger; etc.). Manual FACS-coding by expert raters (van der Schalk et al., 2011; Banziger et al., 2012), and also automated computation (Lucey et al., 2010; Cosker et al., 2011; Mavadati et al., 2013; Zhang et al., 2014), have been applied to dynamic expression databases only on the apex. The estimation at multiple intensities is, however, required because, in real life, expressions vary in intensity, which is often a critical cue to interpret their meaning. Accordingly, we computed the objective evidence of each of six basic expressions and also the evidence of each of 20 AUs, across 31 intensities from neutral (0% intensity) to emotional (100% intensity) in 3.33% intensity steps. This adds to recent work (Calvo et al., 2016; Wingenbach et al., 2016) regarding the role of intensity on the categorization of dynamic expressions. This approach will be particularly useful for expression discrimination studies, e.g., the lowest intensity or threshold at which a particular emotion is recognized and differentiated from others and from neutral faces.

With these two issues in mind, in the current study we developed and validated a dynamic version (KDEF-dyn) of the original KDEF database in static format (Lundqvist et al., 1998), to extend research possibilities. The photographic KDEF stimuli have been validated in large norming studies (Calvo and Lundqvist, 2008; Goeleven et al., 2008), and widely used in behavioral (e.g., Calvo et al., 2013; Sanchez et al., 2014; Gupta et al., 2016) and neurophysiological (e.g., Bublatzky et al., 2014; Calvo and Beltrán, 2014; Adamaszek et al., 2015) research. The original KDEF database has been cited in over 1,980 published articles, according to Google Scholar^1^ (accessed 18.09.2018). We took advantage of this research on the static KDEF stimuli to produce dynamic expressions of 40 different models, each portraying the six basic emotions.

To *develop* dynamic expressions, we applied morphing animation software (FantaMorph, v. 5.4.2; Abrosoft) to the original KDEF photographs. For each encoder and emotion, we created a 1,033-ms video-clip of 31 frames starting with a neutral face and ending with a full-blown emotional face. Thus, we tried to mimic real-life expressions and approximate the average natural speed of emotional expression development from a neutral face, since apex of facial expression is generally reached within 1 s for basic emotions (Pollick et al., 2003; Hoffmann et al., 2010). Admittedly, dynamic morphing creates linear movement, which can make expressions appear as less natural than on-line video recordings. Nevertheless, although non-linear changes are generally judged as more natural than linear motion, morphing does not necessarily compromise naturalness (Cosker et al., 2010, 2015). In fact, dynamically morphed facial expressions have often been employed in prior research on facial emotion recognition, with behavioral (Hoffmann et al., 2010; Fiorentini and Viviani, 2011; Recio et al., 2013; Calvo et al., 2016) and neurophysiological (Popov et al., 2013; Harris et al., 2014; Recio et al., 2014; Vrticka et al., 2014) measures being sensitive to expression manipulations. The morphing technique involves some advantages, such as fine-grained control and standardization of expressive intensity, unfolding speed, and duration. We chose this approach as a balance between (reduced) ecological validity and (enhanced) experimental control.

To *validate* the KDEF-dyn database, we followed two approaches, each with several measures. First, we collected data from *human observers* in an expression categorization task including measures of (a) correct recognition responses, i.e., the probability that they coincided with the intended KDEF expression, (b) reaction times indicating processing efficiency, and (c) the probability of confusions across different expressions, for each of the six basic emotions. Second, with Emotient FACET software (v. 6.1.2667.3; iMotions), we performed *automated facial expression analyses* (Bartlett and Whitehill, 2011; Olderbak et al., 2014; Cohn and De la Torre, 2015; Girard et al., 2015; Dente et al., 2017) of (a) the probability of each expression to be detected, as a function of spatial maps of facial features, and also (b) the probability of each of 20 AUs to be activated, i.e., muscle movements, according to FACS (Ekman and Friesen, 1978; Ekman et al., 2002). The automated analyses of expressions and AUs were performed for 31 intensities (including the neutral baseline) of each emotional facial expression (including apex), while the human recognition measures were obtained for the maximum expressive intensity only. These measures indicate to what extent each KDEF stimulus is consistently categorized, the objective evidence for each facial expression configuration, and the specific morphological features.

The current KDEF-dyn database contributes to existing databases of dynamic facial expression stimuli in several respects. First, the combined validation approach (with both ‘subjective’ human categorization data and ‘objective’ automated assessment data) provides researchers with empirical and theoretical criteria to select stimuli depending on various dimensions (recognition accuracy and efficiency, susceptibility to specific confusions, and automated classification of expressions and AUs). In a dataset file (see Supplementary Dataset S1), each stimulus can be ordered according to each of these measures. Second, due to the standardization of expression unfolding speed and duration for all the stimuli, the present database allows for a fine-grained investigation of emotion recognition as a function of expressive intensity. We provide evidence values from automated analysis of expressions and AUs for each frame of each video-clip. In a dataset file (see Supplementary Dataset S2), such values are shown for each of 31 intensity levels of each stimulus, from 0 (neutral) to 100% (full-blown emotion). Third, another novel contribution involves the control of multiple non-expressive perceptual factors (e.g., low-level image properties) that might otherwise confound expression recognition differences. In a dataset file (see Supplementary Dataset S3), each stimulus has been quantified in terms of such perceptual factors across each of 31 expressive intensity levels. Potential applications and limitations will be considered in the Section “Discussion.”

## Materials and Methods

### Participants

Ninety-six university undergraduates (56 females and 40 males; aged 18–30 years; *M* = 21.2 years) from different courses (Psychology, Medicine, Law, Economics, and Education) participated voluntarily for payment (5 €) or course credit, after signing written informed consent. Four more participants were excluded from the analyses because their mean correct recognition rate was below 50% for three or more expressions. An *a priori* power calculation using *G^∗^Power* (v. 3.1.9.2; Faul et al., 2007) showed that 46 participants would be sufficient to detect a medium effect size (Cohen’s *d* = 0.60) at α = 0.05, with power of 0.98. As this was a norming study of stimulus materials, a larger participant sample was used to obtain stable and representative average scores for each stimulus. The study was approved by the Ethics Committee of University of La Laguna (protocol CEIBA2017-0227), and was conducted in accordance with the Declaration of Helsinki 2008.

### Stimuli

The color photographs of 40 posers (20 females and 20 males) in frontal view from the KDEF database (Lundqvist et al., 1998) displaying six emotional facial expressions (happiness, sadness, anger, fear, disgust, and surprise) were used. The KDEF identities (see Supplementary Dataset S1) were the same as in a previous norming study using photographic stimuli (Calvo and Lundqvist, 2008). For the current study, 240 dynamic video-clip versions (1,033-ms duration) of the original KDEF photographs were constructed. The face stimuli were morphed with FantaMorph (Abrosoft) computer software. For each expression of each poser, we created a 1,033-ms sequence of 31 (33.33-ms) frames smoothly increasing expressive intensity at 30 frames per second (fps), starting with a neutral face as the first frame (frame 0; original KDEF), and ending with an emotional face (happy, sad, etc.) as the final frame (frame 30; original KDEF). Video-clips are shown as supporting information (see Supplementary Dataset S4). A very similar or identical procedure and display duration was used previously (Schultz and Pilz, 2009; Johnston et al., 2013; Wingenbach et al., 2016). Each face stimulus subtended a visual angle of 10.6° (height) × 8° (width) at a 70-cm viewing distance (this approximates the size of a real face, i.e., 18.5 × 13.8 cm, from a 1-m distance).

### Procedure

The 96 participants were presented with all 240 video-clips (40 posers × 6 expressions) in six blocks of 40 trials each, and a short break after each block. Block order was counterbalanced, and trial order and type of expression were randomized within each block. The stimuli were displayed on a computer screen (12-in TFT LED LCD with a 1,366 × 768 resolution) by means of E-Prime 2.0 software. Participants were told that short videos of faces with different expressions would be presented, and were asked to indicate which expression was shown on each trial, by pressing a key out of six, as soon and as accurately as possible, with their dominant index finger. Between trials, the index finger was placed at a predetermined location in the middle of the spacebar, equidistant from all six response keys (from 4 to 9). During the instructions, the six basic expressions were identified, as well as the location of the keys to be pressed for each category. Twelve video-clips of two additional, non-KDEF encoders displaying six emotional expressions served as practice trials.

The sequence of events on each trial was as follows. After an initial 500-ms central fixation cross on a screen, a video-clip showed a facial expression that unfolded for 1,033 ms. Following face offset, graphical instructions appeared on the screen for responding: Six small boxes were arranged horizontally, numbered from 4 to 9, with each box/number associated to a verbal label (e.g., 4: happy; 5: sad, etc.). The assignment of expressions to numbers was counterbalanced across participants. For categorizing each expression, participants pressed one key (from 4 to 9) in the upper row of a standard computer keyboard. The selected response and reaction times (RTs; from the video-clip offset) were recorded. There was a 1,500-ms intertrial interval.

### Design and Measures

We used a within-subjects experimental design, with expressive category (happiness, sadness, anger, fear, disgust, and surprise) as a factor. As dependent variables, we measured hits, i.e., the probability that responses coincided with the displayed expression (e.g., responding “happy” when the face stimulus was intended to convey happiness), and RTs. In addition, we identified the type of confusions, i.e., the probability that each target (the actually displayed expression) was categorized as each of the other five, non-target expressions (e.g., if the target was anger on a trial, the five non-targets were happiness, sadness, disgust, fear, and surprise). These measures, along with those involving automated expression analysis (see below), are provided as supplementary data for each KDEF-dyn stimulus (see Supplementary Dataset S1).

### Automated Facial Expression Analysis

In addition to the human observers’ performance measures, we subjected the video stimuli to automated face analysis by means of Emotient FACET software, which is assumed to detect facial features (e.g., mouth corners) and feature groups, and then to classify the image as belonging to a particular emotional expression category by comparing the resulting output maps with template images. Recently, FACET has been used in psychological and applied research (see Dente et al., 2017). The automated analysis provides two types of measures (see Gordon et al., 2011; Olderbak et al., 2014): (a) *expression* evidence scores for each category: joy, anger, surprise, fear, disgust, sadness, and contempt, in addition to neutral; and (b) AUs evidence scores (for 20 AUs: 1, 2, 4, 5, 6, 7, 9, 10, 12, 14, 15, 17, 18, 20, 23, 24, 25, 26, 28, and 43), according to FACS (Ekman et al., 2002); see also (Cohn et al., 2007; Cohn and De la Torre, 2015). AUs are anatomically related to the movement of specific face muscles (e.g., AU12 involves the contraction of the zygomaticus major muscle, which draws the angle of the mouth superiorly and posteriorly to allow for smiling).

We obtained expression and AU evidence scores for each of 31 frames across the 1,033-ms unfolding, for each poser and expression (see Supplementary Dataset S2). The FACET evidence scores quantify the odds (in decimal logarithmic scale) of each expression or AU to be present in a given face stimulus, and can be transformed into probabilities (*p*) with the formula *p* = 1/(1 + 10^-evidence score^). An evidence score of zero indicates chance level (0.50/0.50). Positive values indicate greater probabilities that a given expression or AU is present, and negative values indicate greater probabilities that an expression or AU is unlikely to be present in the stimulus. All evidence scores above 1 will approach the probability value of 1, and all evidence scores below -1 will approach a 0 probability. This implies that evidence scores (in odds ratios) are more discriminative than probabilities to detect subtle changes, and the former are more suitable for statistical tests because they tend to be normally distributed. The evidence scores ranged in a continuous scale between -12 and 12. We conducted Kolmogorov–Smirnov and Levene’s tests to exam the assumptions of ANOVA regarding normality and homoscedasticity, respectively. Results revealed that most residuals of the evidence scores for expressions and AUs were normally distributed and homoscedastic (for multivariate ANOVA with the evidence scores used as dependent variables and expression category as a fixed factor; see Supplementary Dataset S2).

### Low-Level Stimulus Image Properties

To examine potential physical and perceptual differences among expression categories across the 1,033-ms unfolding display, we computed (with Matlab 7.0, The Mathworks) the following low-level image statistics of each neutral face and the respective emotional faces for each of 31 frames, at consecutive expressive intensity levels, from 0% intensity (i.e., neutral face) to full-blown emotion (i.e., 100% intensity), in 3.33% steps: mean and variance of luminance, RMS or root mean square contrast, skewness, kurtosis, SNR or signal-to-noise ratio, and entropy. Each low-level property was analyzed by means of a (6: Expression Stimulus) × 31 (Intensity Levels) ANOVA. All the measures were sensitive to the effects of intensity, all *F*s(30,7020) ≥ 38.44, *p* < 0.0001, $\eta_{p}^{2}$ ≥ 0.14), but, importantly, the main effect of expression was never significant (all *F*s < 1, except for skewness: *F*(5,234) = 1.51, *p* = 0.19, *ns*; see Supplementary Dataset S3). Accordingly, the face stimuli of the different expressions did not significantly differ in such physical properties. This rules out purely perceptual factors as responsible for the differences observed in categorization performance by human observers or automated facial expression classification (see below).

## Results

We wanted to relate human observers’ performance and automated facial expression analysis, which had to be conducted for each *stimulus*. Further, the study aimed to obtain and provide other researchers with validation measures for each *stimulus* (i.e., KDEF model identity). Accordingly, the statistical analyses were performed on the stimuli as the error term. This means that the recognition performance scores of the 96 participants were averaged for each of the 240 video-clip stimuli, which served as the units of analysis, with an *N* = 40 for each expression category. All the multiple *post hoc* comparisons in the following analyses involved Bonferroni corrections (with a *p* < 0.05 threshold).

### Analyses of Recognition Performance and Confusions by Human Observers

For response *accuracy*, a one-way (6: Expression) ANOVA yielded significant effects, *F*(5,234) = 32.07, *p* < 0.0001, $\eta_{p}^{2}$ = 0.41. *Post hoc* contrasts revealed significantly better recognition of happiness, surprise, and anger, than sadness and disgust, which were recognized better than fear (see Table 1). The correct response *reaction times*, *F*(5,234) = 69.91, *p* < 0.0001, $\eta_{p}^{2}$ = 0.60, were faster for happiness than for any other expression, followed by surprise and anger (which did not differ from each other), and by disgust and sadness (which did not differ from each other), with fear being recognized more slowly than the other categories. Pairwise (Pearson) *correlations* between response accuracy and reaction times for all the expressions showed that reaction times decreased as accuracy increased (Happiness: *r* = -0.67; Surprise: *r* = -0.72; Anger: *r* = -0.78; Sadness: *r* = -0.64; Disgust: *r* = -0.81; Fear: *r* = -0.71; all *p*s < 0.0001; *N* = 40).

**Table 1:** Mean proportion (%) of hits and confusions in human observers’ responses, and reaction times (for hits only) for each target (stimulus) expression.

	Expression response					
Expression stimulus	Happiness	Surprise	Anger	Sadness	Disgust	Fear
Happiness	**98.5^a^**	1.0^b^	0.0^b^	0.0^b^	0.3^b^	0.2^b^
Surprise	2.8^b^	**93.7^a^**	0.1^c^	0.0^c^	0.2^c^	3.2^b^
Anger	0.2^c^	0.8^bc^	**91.7^a^**	1.3^bc^	3.6^b^	2.4^b^
Sadness	0.5^c^	0.7^c^	1.8^c^	**80.7^a^**	5.7^b^	10.6^b^
Disgust	0.1^d^	0.5^d^	13.4^b^	4.7^bc^	**77.8^a^**	3.5^c^
Fear	0.8^d^	18.5^b^	1.1^d^	2.5^d^	8.5^c^	**68.6^a^**
Hits	98.5^a^	93.7^a^	91.7^a^	80.7^b^	77.8^b^	68.6^c^
Hit RTs	868^a^	1,061^b^	1,140^b^	1,253^c^	1,229^c^	1,431^d^

*Within each expression stimulus category (horizontally), scores with different letters across expression response (i.e., on the same line) are significantly different in *post hoc* multiple contrasts (*p* < 0.05, Bonferroni corrected); expressions sharing a letter are equivalent. Boldface for hits in columns*.

For the analysis of *confusions*, a 6 (Expression Stimulus) × 6 (Expression Response) ANOVA was conducted. Interactive effects, *F*(25,1170) = 836.53, *p* < 0.0001, $\eta_{p}^{2}$ = 0.95, were decomposed by means of separate one-way (6: Expression Response) ANOVAs for each expression stimulus. See the mean scores and multiple contrasts in Table 1. Facial *happiness*, *F*(5,195) = 11922.15, *p* < 0.0001, $\eta_{p}^{2}$ = 1, was very unlikely to be confused. *Surprise*, *F*(5,195) = 2952.68, *p* < 0.0001, $\eta_{p}^{2}$ = 0.99, was slightly confused with fear and happiness. *Anger*, *F*(5,195) = 1625.02, *p* < 0.0001, $\eta_{p}^{2}$ = 0.98, was slightly confused with disgust and fear. *Sadness*, *F*(5,195) = 427.46, *p* < 0.0001, $\eta_{p}^{2}$ = 0.92, was confused with fear and disgust more than with other expressions. *Disgust*, *F*(5,195) = 228.31, *p* < 0.0001, $\eta_{p}^{2}$ = 0.85, was confused with anger and sadness, followed by fear. Finally, *fear*, *F*(5,195) = 315.88, *p* < 0.0001, $\eta_{p}^{2}$ = 0.89, was confused with surprise, followed by disgust.

### Automated Assessment of Expressions With FACET

The evidence scores for each expression were subjected to a 6 (Expression Stimulus) × 7 (Expression Response, i.e., the six basic emotions plus neutral) ANOVA. Main effects of expression stimulus, *F*(5,234) = 73.25, *p* < 0.0001, $\eta_{p}^{2}$ = 0.61, and response, *F*(6,1404) = 142.17, *p* < 0.0001, $\eta_{p}^{2}$ = 0.38, and an interaction, *F*(30,1404) = 152.43, *p* < 0.0001, $\eta_{p}^{2}$ = 0.77, emerged. To decompose the interaction, separate one-way (7: Expression Response) ANOVAs were conducted for each expression stimulus. All the expressions were correctly classified (e.g., facial happiness was classified as joy), with target responses being significantly higher (after Bonferroni corrections) than alternative responses (e.g., happiness classified as surprise, etc.), which were assigned negative scores: Facial *happiness*, *F*(6,234) = 636.60, *p* < 0.0001, $\eta_{p}^{2}$ = 0.94; s*urprise*, *F*(6,234) = 150.16, *p* < 0.0001, $\eta_{p}^{2}$ = 0.79; *anger*, *F*(6,234) = 66.31, *p* < 0.0001, $\eta_{p}^{2}$ = 0.63; *sadness*, *F*(6,234) = 61.98, *p* < 0.0001, $\eta_{p}^{2}$ = 0.61; *disgust*, *F*(6,234) = 196.70, *p* < 0.0001, $\eta_{p}^{2}$ = 0.86; and *fear*, *F*(6,234) = 31.44, *p* < 0.0001, $\eta_{p}^{2}$ = 0.45. The interaction reflected the fact that the correct response scores were higher for happy expressions, followed by disgust and surprise (which did not differ from each other), followed by anger, sadness, and fear (which did not differ from one another), as indicated by a one-way (6: Expression Stimulus) ANOVA, *F*(5,234) = 64.34, *p* < 0.0001, $\eta_{p}^{2}$ = 0.58, and multiple *post hoc* comparisons. See the mean scores and contrasts in Table 2.

**Table 2:** Mean raw evidence scores (odds ratios) of each expression (response) for each target (stimulus) expression.

	Expression response						
Expression stimulus	Happiness	Surprise	Anger	Sadness	Disgust	Fear	Neutral
Happiness	**6.4^a^**	-8.6^e^	-7.7^d^	-9.2^e^	-5.4^c^	-3.9^b^	-11.9^f^
Surprise	-5.6^d^	**3.5^a^**	-3.9^c^	-6.1^d^	-4.5^c^	0.8^b^	-4.8^cd^
Anger	-6.3^e^	-4.8^d^	**1.7^a^**	-2.8^c^	-0.4^b^	-2.8^c^	-2.6^bc^
Sadness	-4.6^e^	-4.1^e^	-2.3^d^	**1.7^a^**	-1.3^c^	-0.2^b^	-2.2^cd^
Disgust	-5.4^d^	-8.2^e^	-1.6^b^	-5.7^d^	**4.2^a^**	-3.8^c^	-8.2^e^
Fear	-4.0^d^	-1.3^b^	-2.9^cd^	-3.1^cd^	-2.1^bc^	**1.6^a^**	-4.4^de^
Target	6.4^a^	3.5^b^	1.7^c^	1.7^c^	4.2^b^	1.6^c^	

*Automated analysis computed by Emotient FACET software. Within each expression stimulus category (horizontally), scores with different letters across expression response (i.e., on the same line) are significantly different in *post hoc* multiple contrasts (*p* < 0.05, Bonferroni corrected); expressions sharing a letter are equivalent. Boldface for correct responses to target (stimulus) expressions. Target: correct classification of each stimulus*.

### Automated Assessment of Expressive Intensity With FACET

**FIGURE 1:**
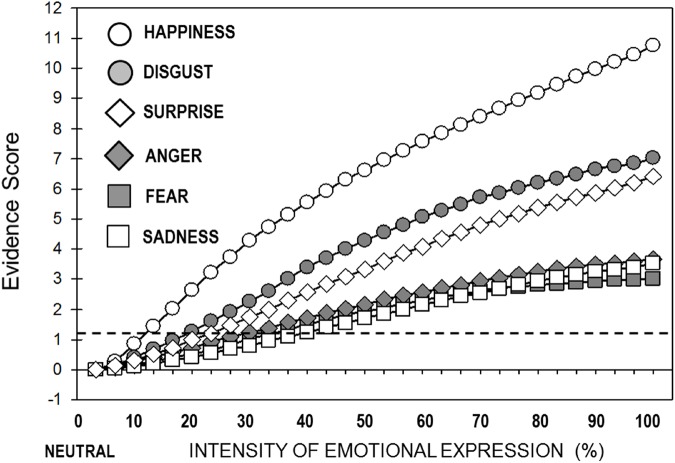
Automated assessment of expressive intensity. Mean automated (FACET) *difference* (emotional minus neutral) evidence scores of each type of expression (response) across intensity levels for each expression stimulus. Above the dotted line: significantly different from the 0% (neutral) baseline (happy: 13.3% of intensity; disgust: 20.0%; surprise: 23.3%; anger and fear: 26.7%; sadness: 36.7%).

To examine expression classification by FACET as a function of expressive *intensity*, we conducted a 6 (Stimulus Expression) × 31 (Intensity Levels: 0% or neutral, 3.3%, 6.7%, etc., and 100% or full-blown emotion) ANOVA on the evidence scores. Effects of expression, *F*(5,7254) = 420.79, *p* < 0.0001, $\eta_{p}^{2}$ = 0.23, intensity, *F*(30,7254) = 593.43, *p* < 0.0001, $\eta_{p}^{2}$ = 0.71, and an interaction, *F*(150,7254) = 23.66, *p* < 0.0001, $\eta_{p}^{2}$ = 0.33, emerged. Separate one-way (Intensity: 31) ANOVAs were performed for each expression to determine the intensity *threshold*, i.e., when significant evidence of each emotion started relative to the neutral face baseline. Facial *happiness*, *F*(30,1209) = 232.76, *p* < 0.0001, $\eta_{p}^{2}$ = 0.85, started to be correctly classified as such at 13.3% intensity (*p* = 0.003, after Bonferroni corrections); *disgust*, *F*(30,1209) = 146.76, *p* < 0.0001, $\eta_{p}^{2}$ = 0.78, at 20.0% intensity (*p* = 0.002); *surprise*, *F*(30,1209) = 109.37, *p* < 0.0001, $\eta_{p}^{2}$ = 0.73, at 23.3% (*p* = 0.012); *anger*, *F*(30,1209) = 43.38, *p* < 0.0001, $\eta_{p}^{2}$ = 0.52, at 26.7% (*p* = 0.02); *fear*, *F*(30,1209) = 52.47, *p* < 0.0001, $\eta_{p}^{2}$ = 0.57, at 26.7% (*p* = 0.039); and *sadness*, *F*(30,1209) = 44.45, *p* < 0.0001, $\eta_{p}^{2}$ = 0.53, at 36.7% intensity (*p* = 0.007). Figure 1 shows the pattern of automated expression classification as a function of expressive intensity.

### Automated Assessment of Action Units (AUs) With FACET

The evidence scores (at 100% intensity of expression) of AUs were subjected to a 6 (Expression Stimulus) × 20 (AUs) ANOVA. Effects of expression, *F*(5,234) = 30.69, *p* < 0.0001, $\eta_{p}^{2}$ = 0.40, AUs, *F*(19,4446) = 433.60, *p* < 0.0001, $\eta_{p}^{2}$ = 0.65, and an interaction, *F*(95,4446) = 100.63, *p* < 0.0001, $\eta_{p}^{2}$ = 0.68, emerged. For all the AUs, there were significant differences across expressions, all *F*s(5,234) ≥ 23.64, *p* < 0.0001, $\eta_{p}^{2}$ ≥ 0.34. Table 3 shows the 100% intensity AU scores.

**Table 3:** Mean raw evidence scores (odds ratios) of action units (AUs) for each expression (100% expressive intensity).

		Expression response					
Action Units		Happiness	Surprise	Anger	Sadness	Disgust	Fear
AU1	Inner brow raiser	-1.11	**1.51**	-1.76	**1.38**	-2.23	**1.58**
AU2	Outer brow raiser	-0.71	**1.93**	-1.75	-0.15	-1.60	0.71
AU4	Brow lowerer	-1.65	-1.08	**1.86**	**1.55**	**1.70**	0.92
AU5	Upper lid raiser	-1.41	**1.99**	0.31	-0.19	-0.97	**1.26**
AU6	Cheek raiser	**2.88**	-2.32	-0.24	-0.28	**1.02**	-0.72
AU7	Lid tightener	0.43	-1.10	**0.89**	0.09	**1.28**	-0.15
AU9	Nose wrinkle	-2.49	-5.22	0.19	-2.45	**3.48**	-3.25
AU10	Upper lip raiser	-0.40	-1.79	0.43	-0.17	**3.55**	-0.34
AU12	Lip corner puller	**4.06**	-1.60	-1.80	-1.04	-1.29	-0.76
AU14	Dimpler	-1.73	-2.62	-1.94	-1.37	-3.58	-1.88
AU15	Lip corner depressor	-1.98	-1.87	-0.99	**0.98**	0.16	-1.22
AU17	Chin raiser	-1.79	-2.53	-0.31	0.25	0.49	-2.02
AU18	Lip puckerer	-9.79	-3.04	-1.69	-2.08	-4.97	-3.83
AU20	Lip stretcher	-0.23	-0.74	-1.48	-0.08	-0.07	0.37
AU23	Lip tightener	-1.58	-1.13	-0.23	-0.80	-0.89	-1.09
AU24	Lip pressor	-2.89	-3.38	-1.07	-0.97	-2.54	-2.77
AU25	Lips part	**2.07**	**2.58**	-1.41	-1.31	0.94	**1.48**
AU26	Jaw drop	-0.05	**2.27**	-2.44	-1.76	-1.82	0.02
AU28	Lip suck	-3.42	-4.84	-3.80	-2.99	-6.07	-3.54
AU43	Eyes closed	-3.45	-0.96	-1.20	-1.47	-0.99	-1.33

*Automated analysis computed by Emotient FACET Software. Boldface: AU evidence scores for emotional faces significantly higher than those for neutral faces *and* above 0. They represent the AUs specifically associated with each expression*.

**FIGURE 2:**
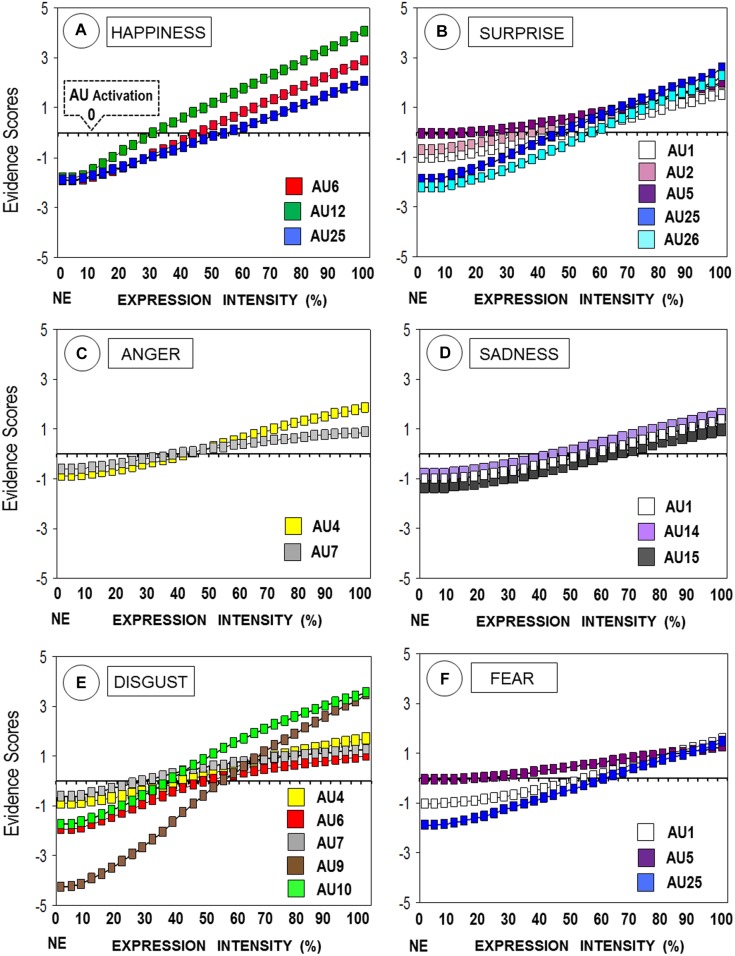
Distribution of AU evidence scores across levels of expressive intensity. For each expression, AUs were selected if scores were higher than for the neutral face (NE, or 0% expression intensity) and were positive and above the 0 AU activation baseline. AU1: inner brow raiser; AU2: outer brow raiser; AU4: brow lowerer; AU5: upper lid raiser; AU6: cheek raiser; AU7: lid tightener; AU9: nose wrinkle; AU10: upper lip raiser; AU12: lip corner puller; AU15: lip corner depressor; AU25: lips part; AU26: jaw drop. **(A)** Happiness; **(B)** Surprise; **(C)** Anger; **(D)** Sadness; **(E)** Disgust; **(F)** Fear.

To interpret the interaction and determine the association of specific AUs to particular expressions, we used two complementary approaches. First, we examined whether, for each AU and emotional expression, the scores were positive and above 0 (thus revealing that an AU was in fact present), by means of *t*-tests for dependent samples. Significant differences appeared for all the AUs in boldface in Table 3, all *t*s(39) ≥ 5.53, *p* < 0.0001, *d* ≥ 0.87. Second, for each AU, we examined whether scores were higher for each emotional expression (at any intensity level from 3.33 to 100%) relative to those for the neutral face, in one-way (31: Intensity level) ANOVAs, followed by Bonferroni (*p* < 0.05) corrections. Significant differences appeared for all the AUs in boldface in Table 3, *F*s(30,1170) ≥ 59.62, *p* < 0.0001, $\eta_{p}^{2}$ = 0.61. Figure 2 shows the variations in the selected AUs (those that fulfilled both criteria, i.e., significantly above 0 and above neutral faces) across expressive intensities. In sum, facial happiness or joy was significantly characterized by AUs 6, 12, and 25; surprise, by AUs 1, 2, 5, 25, and 26; anger, by AUs 4 and 7; sadness, by AUs 1, 4, and 15; disgust, by AUs 4, 6, 7, 9, and 10; and fear, by AUs 1, 5, and 25.

### Relationships Between Human Observers’ Performance (Responses and RTs) and Automated Assessment With FACET (Evidence Scores of Expressions and AUs)

Intra-class correlation (*ICC*, 2) analyses revealed high classification consistency between the automated evidence scores and hits from human raters, separately for each emotional category (*N* = 40; Happiness: *ICC* = 0.93; Surprise: *ICC* = 0.94; Anger: *ICC* = 0.89; Sadness: *ICC* = 0.95; Disgust: *ICC* = 0.76; Fear: *ICC* = 0.65; all *p*s < 0.001; 95% CI). *ICC*s were calculated as consistency between the proportion of hits for each KDEF model (averaged across all 96 human observers) and the evidence scores recalculated into probabilities as *p* = 1/(1 + 10^-evidence score^). Also, RTs for observers’ hits were negatively related to automated evidence of expressions (Happiness: *r* = -0.45; Surprise: *r* = -0.51; Anger: *r* = -0.40; Sadness: *r* = -0.41; Disgust: *r* = -0.58; Fear: *r* = -0.47; all *p*s ≤ 0.01; *N* = 40).

In addition, there were positive correlations between specific AUs and the probability of human categorization responses. Most of the significantly related (all *p*s < 0.0001; *N* = 240) AUs were those that typically characterize each expression: The probability that observers categorized expressions (a) as *happy* was related to AU6 (*r* = 0.67) and AU12 (*r* = 0.90); (b) as *surprised*, to AU1 (*r* = 0.45), AU2 (*r* = 0.73), AU5 (*r* = 0.68), AU25 (*r* = 0.45), and AU26 (*r* = 0.77); (c) as *angry*, to AU4 (*r* = 0.41), AU7 (*r* = 0.37), and AU23 (*r* = 0.48); (d) as *sad*, to AU1 (*r* = 0.36), AU4 (*r* = 0.34), AU15 (*r* = 0.63), and AU24 (*r* = 0.44); (e) as *disgusted*, to AU4 (*r* = 0.36), AU7 (*r* = 0.50), AU9 (*r* = 0.73), and AU10 (*r* = 0.77); and (f) as *fearful*, to AU1 (*r* = 0.42) and AU5 (*r* = 0.34).

## Discussion

We aimed to provide researchers of emotional facial expression processing with a set of useful and valid dynamic stimuli. To this end, with agreed time parameters (i.e., unfolding speed to expressive apex within 1 s; Schultz and Pilz, 2009; Hoffmann et al., 2010; Johnston et al., 2013; Wingenbach et al., 2016), we animated static face stimuli of the KDEF database (Lundqvist et al., 1998). The current study examined the resulting KDEF-dyn video-clip stimuli from two complementary approaches: human observer judgments and automated assessment of facial expression. A variety of measures (recognition accuracy, efficiency, and confusions, as well as automated classification of expressions and detection of AUs as a function of intensity, in addition to low-level image properties) were obtained, and are shown on a stimulus level as supplementary data. They will supply researchers with an instrument to select the stimuli as a function of multiple criteria.

### Recognition Patterns of Static and Dynamic Expressions

Human observers correctly recognized all the expressions (as they were intended) well-above chance level (*M* = 85.2%). Happy faces were recognized better and faster—and fearful faces, less accurately and more slowly—than others, with confusions of fear as surprise, disgust as anger, and sadness as fear. The patterns of recognition accuracy, processing efficiency, and confusions across dynamic expressions converge with those found in prior research for *static expressions*, using different stimulus databases. Regarding *recognition accuracy*, Nelson and Russell reviewed 38 sets of data from 17 studies (Nelson and Russell, 2013): Scores were highest for facial happiness (89%), followed by surprise (83%), which were higher than for sadness and anger (71 and 68%, respectively), followed by disgust and fear (65 and 59%, respectively). This coincides with our own relative differences (see also Tottenham et al., 2009; Recio et al., 2014; Calvo et al., 2016). Such a consistency extends also to *processing efficiency*, as happy faces are typically recognized faster, followed by surprise, while fear is recognized most slowly (Calder et al., 2000; Elfenbein and Ambady, 2003; Palermo and Coltheart, 2004; Calvo and Nummenmaa, 2009). The pattern of *confusions* is also consistent, as they have been found to occur systematically between disgust and anger, and between surprise and fear, and to a lesser extent between sadness and fear (Palermo and Coltheart, 2004; Calvo and Lundqvist, 2008; Tottenham et al., 2009; Recio et al., 2013).

Further validation comes from prior research using *dynamic expression* stimuli. First, three studies included all six basic expressions in dynamic *morphing format* from three different databases. Calvo et al. (2016) presented real faces (24 models of the KDEF-dyn database) for 1 s. Recio et al. (2014) presented real faces (from the RaFD; Langner et al., 2010) for 600 ms. Recio et al. (2013) displayed computer-generated faces (FACSGen 2.0; Krumhuber et al., 2012) for 900 ms. The pattern of recognition accuracy across expressions was similar in all three studies, with happy faces being identified most accurately (also including higher *A’* sensitivity; Calvo et al., 2016), and disgusted and fearful faces, least accurately (and lower *A’* sensitivity; Calvo et al., 2016). In addition, in all three studies, fear was likely to be confused with surprise, disgust with anger, and there was some confusion between sadness and fear. Second, regarding the dynamic stimulus sets based on on-line *video recordings* (e.g., van der Schalk et al., 2011; Banziger et al., 2012; Kaulard et al., 2012; Zhang et al., 2014; O’Reilly et al., 2016; Wingenbach et al., 2016; see the 22 databases reviewed by Krumhuber et al., 2017), it is difficult to make comparisons because some studies did not measure recognition performance (accuracy, RTs, or confusions), and due to considerable variations in number of expressive categories and display times (among many other methodological differences). The study conducted by Wingenbach et al. (2016) was methodologically more similar to our own. Their relative recognition accuracies and the pattern of RTs across the six basic expressions were comparable to those in the current study. Altogether, this empirical consistency validates the current database.

### Automated Assessment vs. Human Observers

Another major source of validation for the current database involves the use of automated facial expression analysis. First, the automated classification of expressions showed discrimination specificity, with the evidence of each expression being significantly greater for the corresponding stimulus category than for the others. Nevertheless, some expressions, especially, happiness, and also disgust and surprise, were classified better than sadness, anger, and fear (see Table 2), which is in total agreement with results obtained with other automated computation algorithms (Lucey et al., 2010). Second, AUs generally discriminated between expressive categories, and this was in accordance with FACS proposals (Ekman et al., 2002; Olderbak et al., 2014). Some AUs characterized expressions more specifically or strongly than others (see Table 3), e.g., AU12 for happiness, AU25 for surprise, AUs 9 and 10 for disgust, AU1 for fear, and AU4 for anger and sadness (the AU4 combination with other AUs allowed for a clear discrimination between these two expressions; see Table 2). A related pattern has been obtained with different automated AU detection systems (Lucey et al., 2010; Mavadati et al., 2013; Zhang et al., 2014). Third, automated expression classification and also AU evidence scores increased significantly across 3.33% expressive intensity steps between a neutral and an emotional face (see Figures 1, 2). The steepness of such a progressive increase as a function of intensity varied for different expressions and AUs. This approach and results regarding intensity represent a novel contribution and further validate the current video-clip stimuli.

Fourth, importantly, significant correlations emerged between human observers’ performance and automated evidence of expressions (large effect sizes: Cohen’s *d*s ≥ 1.71) and AUs (medium to large effects: *d*s ≥ 0.72). This has implications for expression recognition theories concerning the type of information that is processed and the cognitive processes involved. Computational models such as EMPATH (Dailey et al., 2002, 2010) and support vector machine (SVM) based techniques (Susskind et al., 2007)—and, presumably, FACET—simulate face processing and expression recognition in humans. In these models, facial expressions are computed by “emotionless machines” on purely perceptual grounds, i.e., physical image properties (the morphological structure of facial configurations and the visual saliency of distinctive facial cues), in the absence of affective processing. Accordingly, the fact that the automated classifications of expressions converged with human observers’ judgments in the current study suggests that human expression recognition also relies to a significant extent on the perceptual (devoid of affect) analysis of facial features. Nevertheless, first, while this may be true for photographs or videos of faces, the role of human affective processing is probably greater in actual face-to-face social encounters, when emotional significance becomes relevant for adaptive purposes. Second, it is likely that the morphological facial features of expressions have become associated (through practice) with their affective significance, and thus both would be processed in tandem, therefore explaining the observed correlations.

### Applications and Limitations

The KDEF-dyn database aims to extend the research possibilities of dynamic facial expression stimuli. First, regarding *experimental control*, all the stimuli are equated in multiple image properties that are non-specific of expression—but can act as confounds—(luminance, signal-to-noise ratio, size, orientation, etc.), in addition to standardization of dynamic properties (unfolding speed and duration). Such controls will be particularly useful for neurophysiological and eyetracking research, where the dependent measures are especially sensitive to physical stimulus factors; and also useful for paradigms in which the stimuli must be presented briefly, where display duration needs to be strictly comparable for the different stimuli. A second benefit is related to the role of *expressive intensity*. Instead of considering only the apex, we have established the assessment of expressions and AUs at fine-grained intensities. This is important, as intensity is often critical to interpret the meaning of expressions. By knowing the evidence for each expression and AU at each intensity level, and the time-intensity correspondence in the video-clips (as shown in Supplementary Dataset S3), researchers can easily manipulate the display time of the stimuli to investigate the desired intensity (e.g., by cutting, masking, or stopping each video-clip at the respective time point). This approach will be useful for the investigation of visual processing, particularly for studies of expression discrimination thresholds. A third promising application is concerned with the use of these stimuli in the investigation of *cognitive biases* (attentional and interpretative) in *psychopathology*. For example, it has been shown that individuals with clinical levels of social anxiety are especially prone to detect negatively valenced dynamic expressions at low intensities (Gutiérrez-García and Calvo, 2016, 2017; Gutiérrez-García et al., 2018). A reason for the usefulness of this application to psychopathology research is that dynamic information improves identification of facial affect, particularly for lower intensity and subtle stimuli (Krumhuber et al., 2013), which would increase sensitivity for individuals that are hypervigilant to threat and incongruities in facial expressions.

Researchers should, nonetheless, be aware of potential limitations. First, although standardization of unfolding *speed* is beneficial for experimental control, it can reduce the natural speed variance across expressions. For example, we averaged the 1-s unfolding speed from neutral baseline to emotional apex for all the expressions (see Schultz and Pilz, 2009; Johnston et al., 2013; Wingenbach et al., 2016). However, facial surprise is considered as most natural when it unfolds at a fast pace while sadness is judged as more realistic when the facial expression changes slowly (Sato and Yoshikawa, 2004; Adamaszek et al., 2015). To remedy this potential limitation, it is possible to slow down or speed up the video-clips, by means of video-editing software. Second, we used *posed* instead of spontaneous expressions. The majority of extant dynamic stimulus sets, in fact, include posed expressions, either in response to instructions to perform facial actions or as the enactment of emotional scenarios (van der Schalk et al., 2011; Banziger et al., 2012; Kaulard et al., 2012; O’Reilly et al., 2016; Wingenbach et al., 2016), although some have included spontaneous expressions (Mavadati et al., 2013; Zhang et al., 2014). Posed expressions may lose naturalness and their recognition rates may be inflated, although the former avoid the ambiguity of spontaneous expressions. Third, we used *morphed* expressions. Morphing creates linear movement where all the facial components change at the same time and speed, whereas natural expressions appear to change in a non-linear manner. However, some studies indicate that natural expressions look smooth, uniform, and ballistic (Weiss et al., 1987; Hess et al., 1989), thus actually sharing properties with morphed dynamic expressions. Further, in the current study, automated assessment revealed specificity and sensitivity to expressions and also to AUs in accordance with FACS proposals. This suggests that the possible reduction of naturalness was not critical (see Cosker et al., 2010, 2015).

## Conclusion

We present a set of dynamic facial expressions (KDEF-dyn) based on a widely used database of static expressions (KDEF). The new stimuli have been validated by means of several measures from two approaches: expression categorization by human observers and automated analysis of facial expressions and AUs with computer software. Results show good convergence with prior research using static and dynamic expression stimuli. Although not devoid of limitations, this convergence reinforces the validation of the current database, while offering additional advantages: (a) the use of automated facial expression and AU analysis, with significant correlations between human and automated performance; (b) the control of perceptual properties (e.g., size and multiple low-level image statistics) and stimulus dynamic properties (e.g., duration and unfolding speed); and (c) the systematic and fine-grained gradation of expressive intensities of an otherwise relatively large sample of encoders. This will be useful for behavioral, computational, and neurophysiological studies investigating facial expression processing.

## Availability of Data

The KDEF-dyn stimuli and datasets are freely available for scientific purposes, and can be downloaded from http://kdef.se/versions.html (KDEF-dyn I).

## Author Contributions

MC and DL conceived and designed the experiments. AF-M prepared the materials, performed the experiments, and conducted the statistical analyses. MC wrote the first draft of the manuscript. MC, AF-M, GR, and DL wrote sections and revised the whole manuscript.

## Conflict of Interest Statement

The authors declare that the research was conducted in the absence of any commercial or financial relationships that could be construed as a potential conflict of interest.

## Footnotes

1 https://scholar.google.com/scholar?cites=93971208802805184&as_sdt=2005

## Supplementary Material

DATASET S1 Human and automated expression and action unit categorization. Measures of recognition performance by human observers and automated analysis for each KDEF-dyn stimulus.

DATASET S2 FACET assessment of intensities. Evidence values for each expression and action units, at each of 31 intensity levels (in 3.33% steps).

DATASET S3 Low-level image statistics of intensities. Image values at each of 31 intensity levels (in 3.33% steps).

DATASET S4 Stimuli. Video-clip stimuli_MP4. Two hundred and forty video-clips, separated for each of six emotional expression categories (40 video-clips each).
